# The Efficacy of Antibiotic-Loaded Calcium Sulfate Beads (Stimulan) in Patients with Hip Arthroplasty Infections

**DOI:** 10.3390/jcm13144004

**Published:** 2024-07-09

**Authors:** Florentin Dimofte, Cristina Dimofte, Sorin Ungurianu, Cristina Serban, George Țocu, Nicoleta Cârneciu, Iulia Filip, Laura Bezman, Bogdan Mihnea Ciuntu, Irina Mihaela Abdulan, Raul Mihailov, Radu Dan Necula, Florin Lucian Sabou, Dorel Firescu

**Affiliations:** 1 General Surgery Clinic, “St. Apostol Andrei” County Emergency Clinical Hospital, Strada Brăilei 177, 800578 Galati, Romania; florentin.dimofte@ugal.ro (F.D.); cristina.serban@ugal.ro (C.S.); george.tocu@ugal.ro (G.Ț.); nicoleta.carneciu@ugal.ro (N.C.); iulia.filip@ugal.ro (I.F.); 2 Department of Orthopedic, Faculty of Medicine, “Dunarea de Jos” University of Medicine and Pharmacy, 800008 Galati, Romania; sorinungurianu@yahoo.com; 3 Department Radiology, “Saint John” Emergency Children Hospital, Str. Gheorghe Asachi, Nr.2, 800487 Galati, Romania; cristina.dimofte90@yahoo.com; 4 Department of General Surgery, Faculty of Medicine, “Dunarea de Jos” University of Medicine and Pharmacy, 800008 Galati, Romania; raul.mihailov@ugal.ro (R.M.); dorelfirescu@yahoo.com (D.F.); 5 Department of Laboratory Medicine, Faculty of Medicine, “Dunarea de Jos” University of Medicine and Pharmacy, 800008 Galati, Romania; 6 Department of Ophtalmology, Faculty of Medicine, “Dunarea de Jos” University of Medicine and Pharmacy, 800008 Galati, Romania; laura.bezman@ugal.ro; 7 Department of Anesthesia, Faculty of Medicine, “Dunarea de Jos” University of Medicine and Pharmacy, 800008 Galati, Romania; 8 Department of General Surgery, “Grigore T. Popa” University of Medicine and Pharmacy, 700115 Iași, Romania; 9 Department of Medical Specialties I, “Grigore T. Popa” University of Medicine and Pharmacy, 700115 Iași, Romania; 10 Department of General Surgery, “Transilvania” Faculty of Medicine, 500019 Brașov, Romania; ortomed.bv@gmail.com (R.D.N.); drsabou@yahoo.com (F.L.S.)

**Keywords:** stimulan, calcium sulfate, wound infection, hip arthroplasty, biocomposite

## Abstract

**Background:** Given the increasing rate of infections following hip arthroplasty, one of the current options is the application of a biocomposite at the site of the infection. One of the products used is Stimulan, a completely resorbable calcium sulfate, designed to treat acute and chronic infections. This biocomposite has a controlled purity, is easy to mix with liquid, powder, and antibiotics, and can be applied directly to the site of infection, or it can be injected. **Methods:** We analyzed data from 76 patients, who were admitted to the County Clinical Hospital of Emergency “St. Apostol Andrei” in Galati during January 2017–September 2023, with a diagnosis of infection associated with hip arthroplasty. **Results:** In 69.73% of cases (52 patients), we decided to keep the implant in place. In this subgroup, Stimulan was applied in 26 cases (57.78%). Of these, 100% were cured—compared to the subgroup in which Stimulan was not applied, where this percentage was lower. All patients underwent chemical and mechanical toileting, and for 24 patients (20.27%), it was necessary to ablate the implant with or without the application of Stimulan, then reimplantation of the prosthesis. **Conclusions:** The patients with Stimulan had a longer average length of hospitalization, almost half of them required intervention in two periods, and a quarter required the implantation of spacers. However, the cure rate was higher, only in three people could we not control the infection, and there was no death. This study confirms the effectiveness of the treatment when using a biocomposite in addition to the classical treatment for both acute and chronic cases.

## 1. Introduction

Since hip arthroplasty has become a routine intervention in most medical institutions, the number of infections associated with this intervention is also increasing.

A deep periprosthetic joint infection (PJI) is one of the most dangerous postoperative consequences that might happen. The risk of developing PJI is typically estimated to be around 1%. However, there is significant variability in this statistic across different studies, with reported rates ranging from 0.57% to 2.23% [1]. Even though the likelihood of infection may seem relatively low, the consequences and long-term effects can be devastating.

Microorganisms may defeat the host’s immune system by infecting any prosthetic material in the body. Bacteria situated on the surface of the prosthetic material form structures known as biofilms that adhere to a prosthesis’ surface [2].

Under certain circumstances, germs may enter the joint cavity through the bloodstream or during the placement of a prosthesis. The mouth cavity is a common source of microorganisms, particularly in those who have dental problems. Certain pathogenic bacteria, such as coagulase-negative staphylococci, can occasionally have low virulence and persist because of the biofilm and the prosthetic material, inducing a low-grade, persistent illness with few systemic symptoms. However, infections caused by highly pathogenic organisms, including *Staphylococcus aureus*, might result in septicemia or bacterial endocarditis [3].

The available treatment options for PJI after THA (total hip arthroplasty) include one-stage revision, two-stage revision, and debridement with antibiotics and implant retention (DAIR). Among these options, DAIR is considered the least invasive for surgical PJI management. It is commonly used for acute infections without complicating factors such as significant comorbidity or loosening of the prosthesis. Previous studies have shown that DAIR can lead to shorter hospital stays, lower costs, and improved functional outcomes [4]. However, the effectiveness of DAIR for PJI treatment remains uncertain, as reported failure rates in the literature vary widely, ranging from 0% to as much as 84% [5,6]. It varies depending on factors such as the duration of infection, the host’s immunity, the virulence of the causative microorganism, and the technique used [2]. Due to the poor vascularity and the formation of biofilm, infected artificial joints often do not respond well to systemic antibiotic treatment. Once the biofilm is formed, more aggressive surgical techniques are used.

Considering these aspects, the prompt and efficient treatment of infections occurring after the insertion of prostheses is a priority.

Stimulan, a fully resorbable calcium sulfate, is used to treat acute and persistent infections. This biocomposite has a low drainage level, controlled purity, and is simple to combine with liquid, powder, and antibiotics. It comes in the shape of granules in various sizes that are meant to be applied directly to the infection site, but it can also be injected or used as a paste. When combined with one or more antibiotics, the product’s hydrophilic qualities and choices for standard or fast curing times make it simple to use and flexible enough to adjust as needed [7]. As we mentioned before, the classical treatment recommends local cleaning, mechanical and chemical debridement, intravenous antibiotic treatment, and maintaining or replacing the implant, depending on the case: in acute cases, it is attempted to keep the implant, while in chronic cases, the implant is replaced.

We attempted to maintain the implant in all cases where it was possible. For this purpose, we decided to add a local antibiotic biocomposite to the classic treatment to more effectively treat the infection.

This study aimed to evaluate the effectiveness of the combined treatment, both in acute infections and chronic infections.

## 2. Materials and Methods

### 2.1. Study Design and Setting

We conducted a monocentric, retrospective, cohort study between January 2017 and September 2023 in the County Clinical Hospital of Emergency “St. Apostol Andrei” in Galati. Our research included patients admitted for hip arthroplasty.

### 2.2. Study Participants

From a total of 4244 hip arthroplasty surgeries, 76 patients were enrolled in this study during the mentioned period. The inclusion criterion was the presence of infection associated with hip arthroplasty.

Data were collected on age, gender, laboratory results, intervention type, evolution, and post-surgery outcomes.

### 2.3. Diagnostic

The diagnosis of periprosthetic infection (pathogen which is isolated by culture from at least two separate samples of tissue or fluid obtained from the affected endoprosthetic joint) was established using the criteria published by Kallala et al. [8]:

- Increased erythrocyte sedimentation rate (ESR);
- Increased serum level of C-reactive protein;
- Increased number of leukocytes in the synovial fluid;
- Increased percentage of neutrophils in the polymorphonuclear leukocyte (PMN) synovial fluid;
- The presence of pus in the involved joint;
- Isolation of a microorganism in a single culture of tissue or periprosthetic fluid or more than five neutrophils per microscopic field.

Patients who had a score of at least 4 points out of a possible 6 were considered to have a periprosthetic infection.

The staging of this complication was carried out according to the time interval elapsed since the primary intervention, using the classification proposed by Pellegrini et al., which defines periprosthetic infections as follows [9]:

- Early, appearing less than 3 months post intervention;
- Delayed, appearing between 3 and 24 months post intervention;
- Late, appearing after 24 months post intervention.

### 2.4. Surgical Procedure

The surgical procedures performed were of the DAIR or two-stage exchange type, including surgical debridement, abundant lavage, and the application of 20 cc Stimulan pearls mixed with antibiotics, simultaneously with the intravenous administration of antibiotics.

For the DAIR surgical intervention, we performed a thorough surgical debridement, abundant lavage, keeping the implant in place, the application of Stimulan, and the administration of injectable antibiotics for 6 weeks after the diagnosis of the infection.

For the two-stage surgical intervention, we conducted thorough surgical debridement, extensive washing, removal of the initial implant, and placement of a monobloc spacer made of acrylic acid and Gentamicin (6 mL), along with the application of Stimulan. Injectable antibiotics were administered 6 weeks after diagnosing the infection, and then the final reimplantation of the prosthesis was performed. 

All patients received preoperative antibiotic therapy, a treatment that was extended 6 weeks after the intervention.

We used 20 cc of Stimulan Rapid Cure paste to produce 50 cc of pearls. In a sterile bowl, we combined 2 g of finely ground Vancomycin with the Stimulan powder and mixed until smooth. Additionally, we added 12 mL of Gentamicin and 2 mL of sterile solution from the Stimulan package. We mixed the ingredients for 30 s at a constant temperature of 18 degrees (typical operating room temperature), resulting in the formation of a paste. This paste was then spread onto a silicone pad to create pearls, and it took about 5 min for the paste to harden. Finally, we placed the pearls in the prosthesis chamber, both in the lower part of the acetabulum and as deeply as possible in the upper part.

Post-interventional follow-ups were performed on the third postoperative day. Later, if the patients’ condition allowed, they were discharged 5 days after the surgery with the recommendation to continue the antibiotic treatment for up to 6 weeks. They came for check-ups postoperatively at 14 days, 6 weeks, 3 and 12 months, and then annually.

### 2.5. Ethical Approval

In order to be included in this study, all the patients completed an informed consent form. The ethics approval was received in 2022 (no. 33/23.12.2022 issued by the Ministry of National Education—Faculty of Medicine, “Dunărea de Jos” University of Medicine and Pharmacy, Galati, Romania).

### 2.6. Statistical Methods

SPSS 20.0 (Statistical Package for the Social Sciences, Chicago, IL, USA) was used for data analysis. The Shapiro–Wilk test was used to determine the distribution of continuous data, which were entered as the mean ± standard deviation, or a number representing a percent frequency for continuous variables with a normal distribution. Independent samples were used to compare continuous variables with normal distributions using Student’s *t*-test in the case of two samples. Nonparametric tests were used to evaluate continuous variables that did not satisfy the assumption of normality, such as the Mann–Whitney U test in the case of two samples. Results with a *p*-value < 0.05 were deemed statistically significant.

## 3. Results

From a total of 4244 patients admitted for hip arthroplasty, 76 were diagnosed with an infection associated with the surgical intervention. The annual infection rate following the primary arthroplasty surgical intervention, reported as a percentage of the number of surgical interventions during the period 2017–2023, was as follows: 0.83%, 0.66%, 0.87%, 1.29%, 1.08%, 1.44%, and 1.22%, in the last two years, doubling the number of hip arthroplasty performed in our hospital.

The average age was 67.84 ± 12.04; 65.79% of the patients were men and 34.21% were women. Most of the patients were overweight or obese and had delayed or late infection. The complete characteristics of the group are illustrated in Table 1.

**Figure 1:**
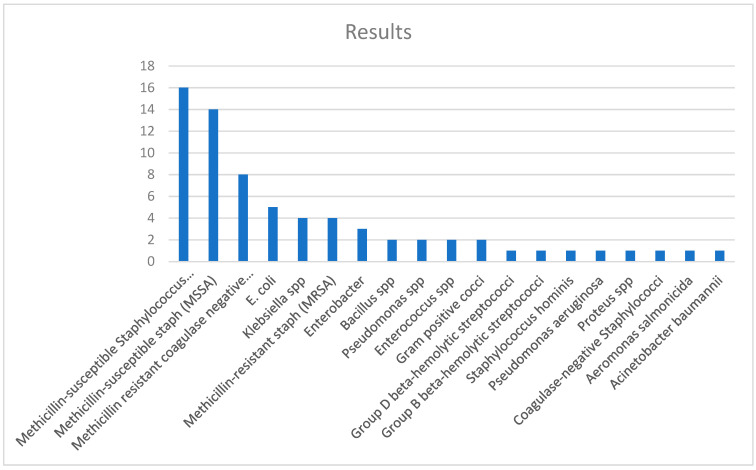
Results of microbial cultures.

All patients were treated according to the antibiogram. The results are summarized in Figure 1.

In the current study, the most frequent pathogen identified was Methicillin-susceptible *Staphylococcus aureus* (MSSA), present in a percentage of 21% (16) of cases from the total group, closely followed by Methicillin-susceptible coagulase-negative Staphylococcus at 18% (14 cases).

For the first-mentioned bacteria, the first-line treatment was Oxacillin 1.5–2 g i.v., 4–6 h in continuous infusion, and Vancomycin i.v. 15–20 mg/kg every 12 h. As alternatives, we used Rifampicin p.o. 600 mg/day or Clindamycin i.v. 600–900 mg/day every 8 h. The duration of treatment was 6 weeks.

For the second one, we used Vancomycin 15–20 mg/kg every 12 h and Levofloxacin 500–750 mg p.o./24 h plus Rifampicin 600 mgp.o./day, the duration of treatment was also 6 weeks.

The following were also identified with increased frequency: Methicillin-resistant coagulase-negative staphylococcus (11%—eight cases), *E. coli* (7%—five cases), Methicillin-resistant staphylococcus aureus (5%—four cases), and *Klebsiella* spp. (5%—three cases).

Among the rare pathogens encountered, we list Pseudomonas aeruginosa, one case, where we administered Meropenem 1 g i.v. every 8 h and Ciprofloxacin 750 mg p.o. every 12 h or 400 mg i.v. every 8 h. The duration of treatment was 6 weeks.

In the study group, most of the infections were mono-bacterial (96%), but we identified three cases with multiple germs: *Staphylococcus aureus*/Citrobacter, *Klebsiella* spp./Glucose non-fermentative Gram-negative bacilli, and *Klebsiella* spp./Methicillin-susceptible *Staphylococcus aureus*.

A small percentage, 8.88% (four cases), had negative cultures, the most likely explanation being self-administration of antibiotics at home before admission to the hospital.

We used Stimulan both for acute and chronic infections.

In our study, the patients who underwent surgery in two stages (two-stage exchange) benefited from the extraction of the primary prosthesis, its replacement with a spacer, and the application of calcium granules mixed with antibiotics. The reimplantation of the prosthesis was performed later.

Although there were no statistically significant differences, the following aspects are worth mentioning when we compared the group with Stimulan versus the one in which we did not use it: the patients with Stimulan had a longer average length of hospitalization; almost half of them required intervention in two periods and a quarter required the implantation of spacers. However, the cure rate was higher, only in three people could we not control the infection, and there was no death (Table 2).

The deceased patients were in the age group of 60–79 years, had comorbidities, and developed MOSF (Multiple Organ System Failure), being hospitalized in the ICU (Intensive Care Unit), where they died.

DAIR. Keeping the implant was decided in 70% of cases. In this subgroup, Stimulan was applied in 26 cases (57.78%). Of these, 93% (42 patients) were cured. Meanwhile, in the subgroup in which Stimulan was not applied, from a total of 26 cases, only 10 (32.25%) were cured.

From the group of patients where Stimulan was not applied, 13 patients presented a recurrence of the infection, and 8 patients died because they had multiple associated comorbidities.

TWO-STAGE EXCHANGE. All patients (24) underwent chemical and mechanical toileting, and for 13 patients (representing 17.10%), it was necessary to ablate the implant with or without the application of Stimulan, and then perform reimplantation of the prosthesis. From this subset, a spacer was applied for 13 patients, representing 17.10%.

For patients who underwent chemical and mechanical surgical toileting, the implant was extracted, and a spacer was added in 13 cases. Stimulan was used for 12 patients with a cure rate of 100%.

**Figure 2:**
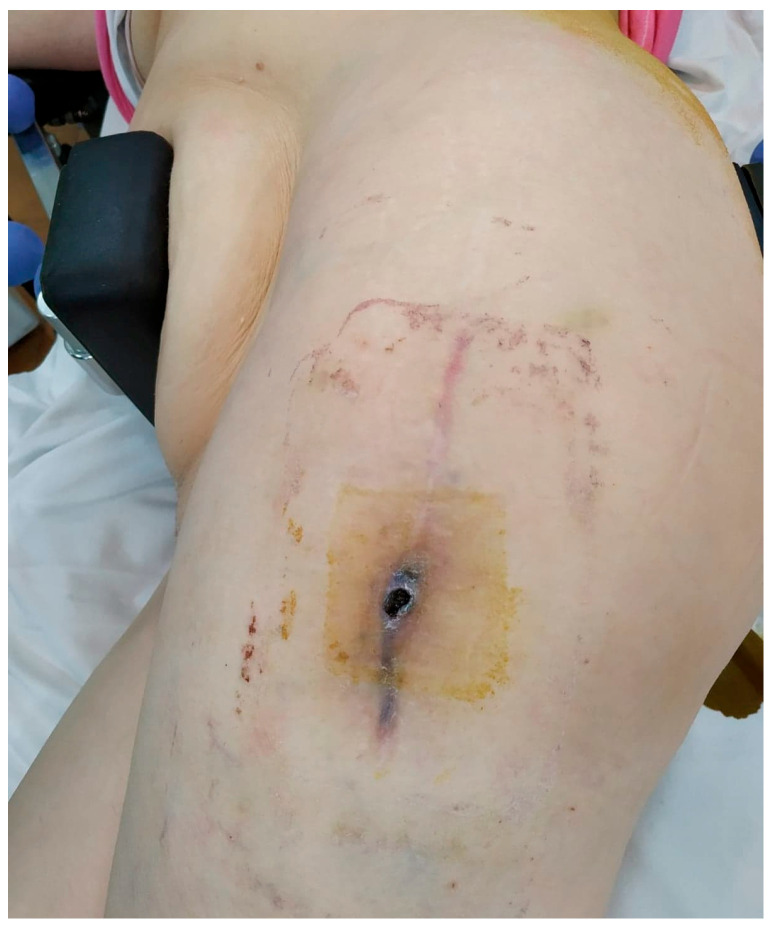
Preoperative aspect of the fistula.

**Figure 3:**
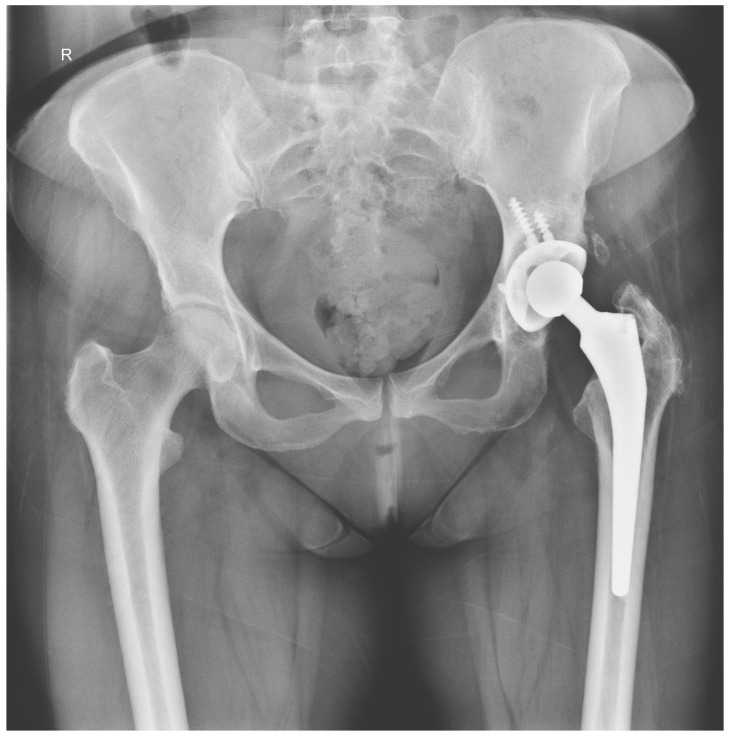
X-ray of pelvis, total left hip Arthroplasty.

**Figure 4:**
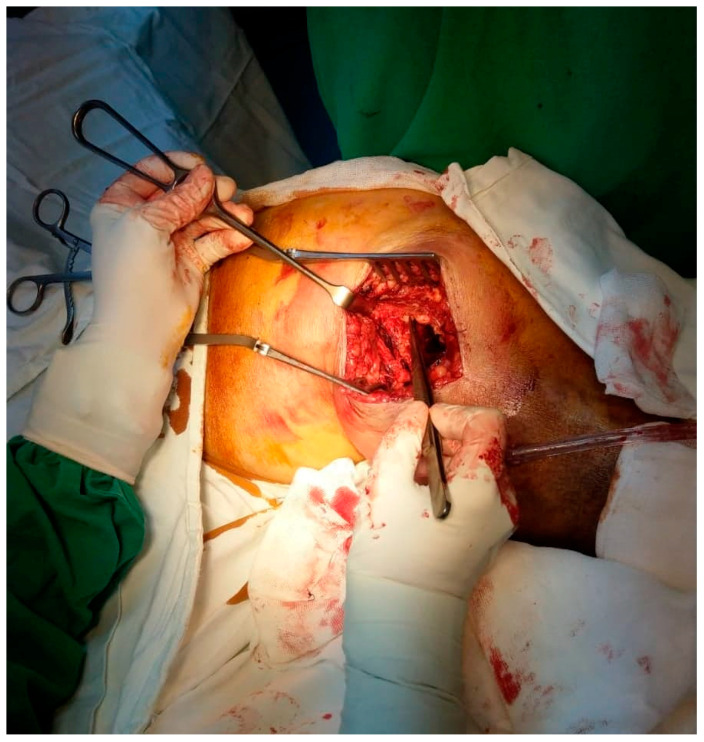
Intraoperative aspect, fitting of the Stimulan granules.

**Figure 5:**
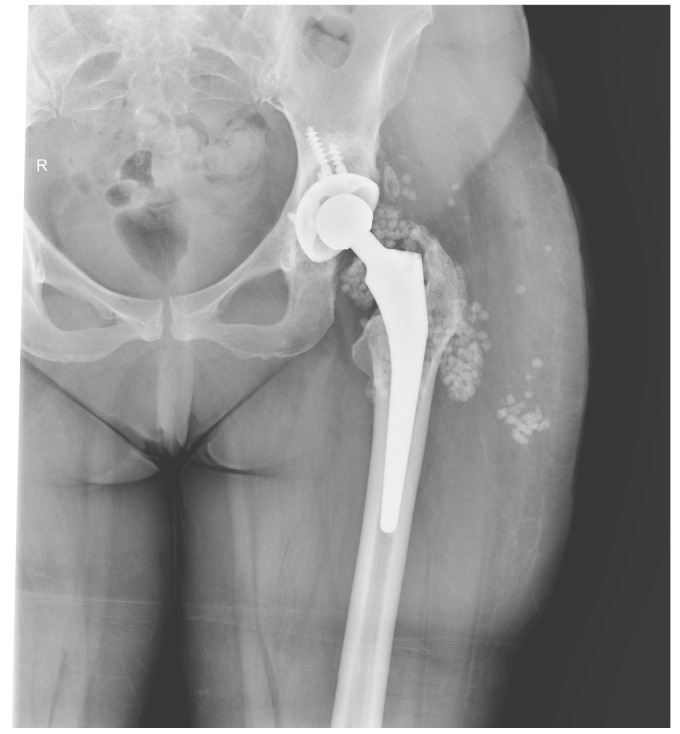
Postoperative pelvis radiography, Stimulan granules present in the left hip.

**Figure 6:**
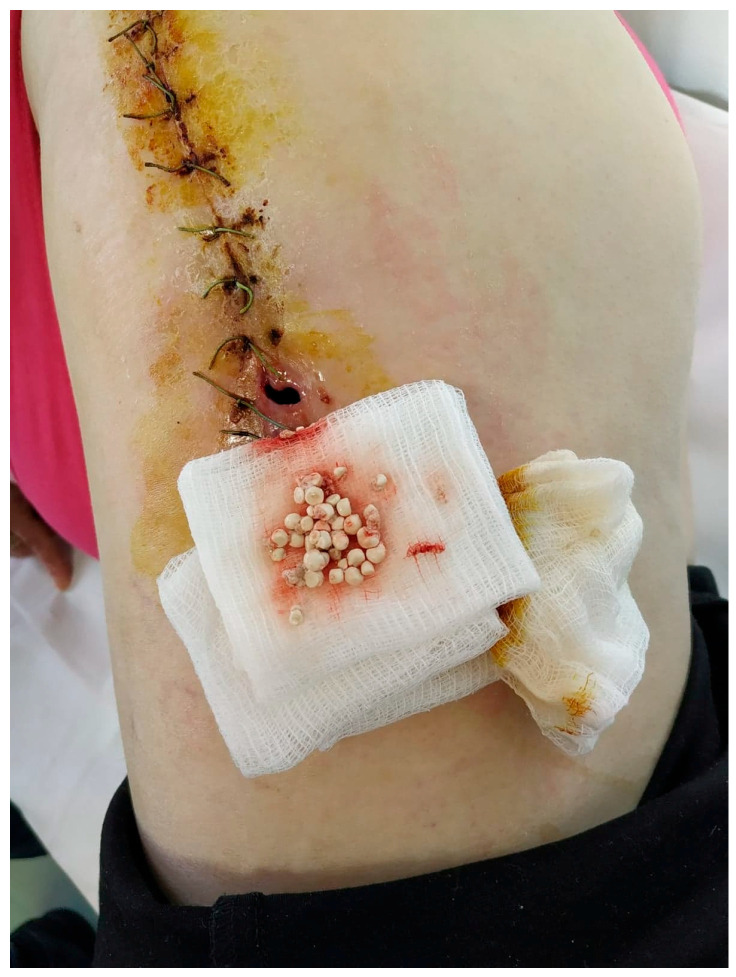
Fourteen days postoperative aspect, externalization of Stimulan granules.

**Figure 7:**
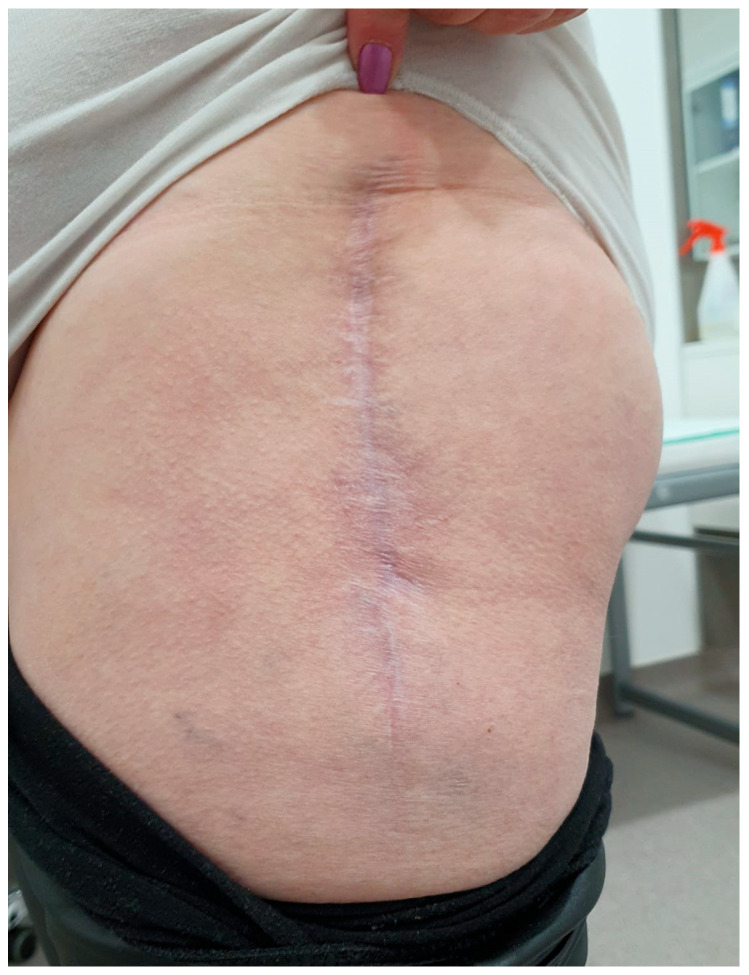
Local aspect of postoperative scar after wound healing.

We did not encounter heterotopic ossification or postoperative hypercalcemia. Instead, we encountered a single postoperative complication 14 days after the surgical intervention with the externalization of Stimulan pearls through the postoperative wound (Figure 2, Figure 3, Figure 4, Figure 5 and Figure 6). After cleaning and removing the externalized pearls and mounting a negative pressure kit for up to 21 days, the skin defect closed with a good evolution both locally and generally (Figure 7).

**Figure 8:**
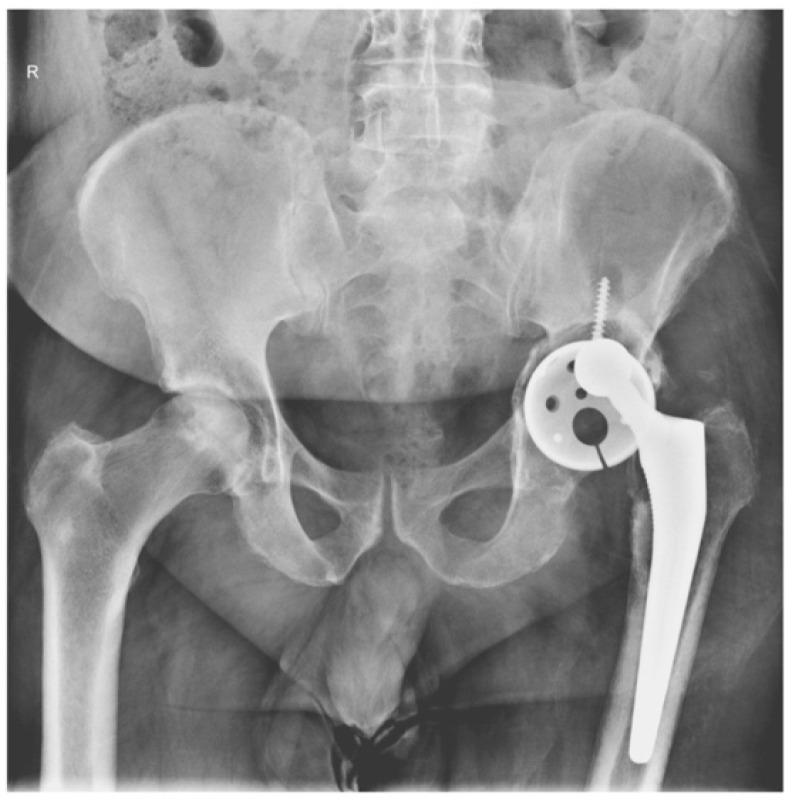
Septic loosening of the acetabular component.

**Figure 9:**
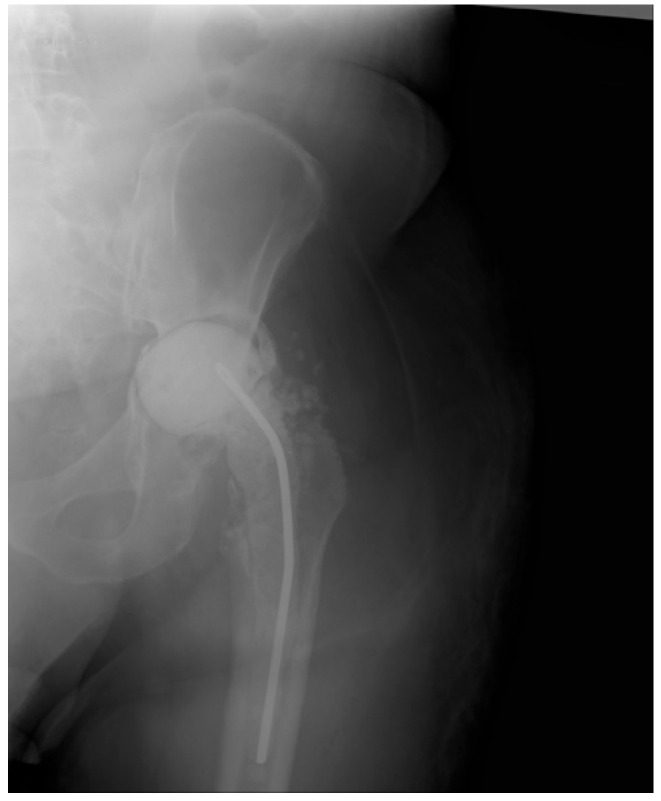
Left hip spacer with Stimulan granules.

**Figure 10:**
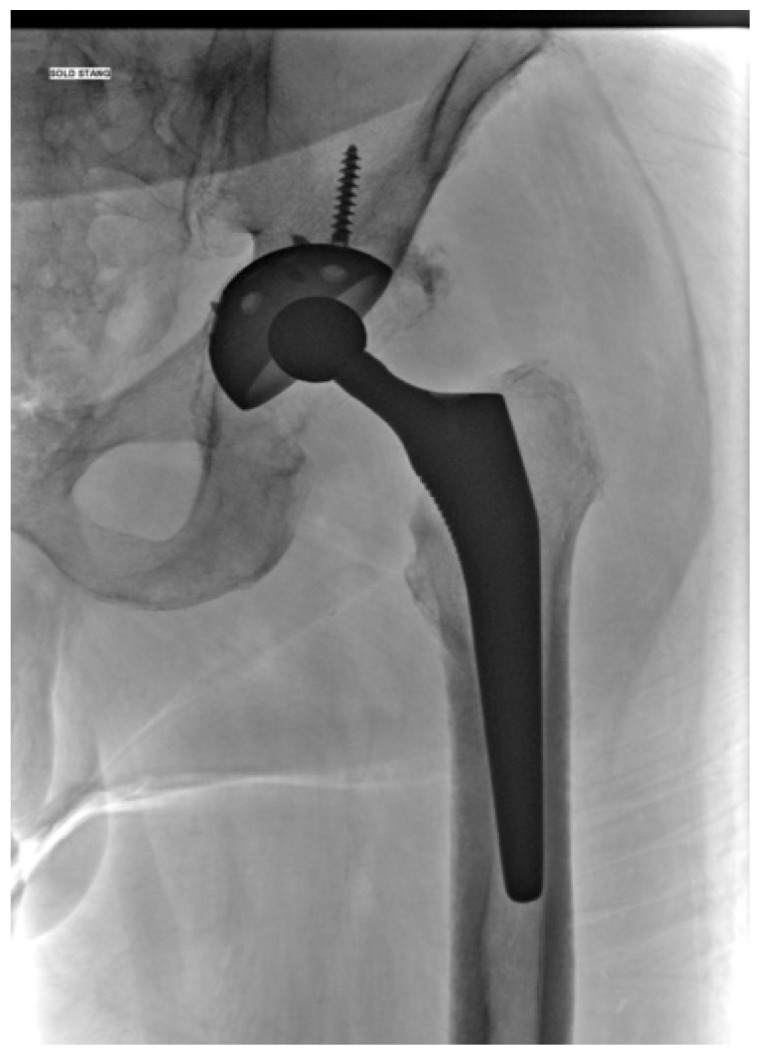
Final prosthesis implantation.

The following images depict a case that was treated in two-stage exchange. In Figure 8, we can observe a septic loosening of the acetabular component, where surgical intervention involved thorough chemical and mechanical debridement, lavage, removal of the primary prosthesis, and application of an antibiotic-loaded spacer augmented with Stimulan granules (Figure 9). Figure 10 shows the reimplanted prosthesis after the two-stage surgical treatment, including the application of Stimulan. The evolution was favorable.

## 4. Discussion

We have examined the current literature on the outcomes of exchange arthroplasties, which primarily focused on reinfection rates and functional outcomes. As suggested by Tsukayama and his colleagues in 1996, the timing of the infection is crucial in determining the appropriate treatment [10]. Therefore, an acute postoperative infection occurring within a month of the initial procedure is typically treated with irrigation and debridement. However, this approach has a higher failure rate for late infections due to bacterial biofilm formation on the prosthesis after this time frame. As a result, delayed infections require the replacement of the prosthesis to effectively control the infection [11].

Lima et al. show in their study that in chronic infections, it is recommended to retain the primary implant and use spacers for some time concurrently with antibiotic therapy and that the treatment of infections through one-stage exchange interventions should be chosen with great care [12]. Following our study on chronic infections, despite careful surgical debridement and abundant irrigation, we applied Stimulan in 65% of cases and achieved a cure in all cases.

In the specialized literature, we can highlight a meta-analysis conducted by Abdulbaset et al. They analyzed 76 articles, but only 5 met the criteria for their study. They noted the effectiveness of this calcium sulfate loaded with high doses of antibiotics applied to the site of infection, but also the occurrence of complications when these doses of antibiotics were administered in the subcutaneous layers. However, their study was not based on much evidence to demonstrate the superiority of this treatment [13].

Complications have been noted. The occurrence of heterotopic ossification, prolonged wound drainage, and hypercalcemia has been reported at rates of 1.1–1.8%, 0.8–3.2%, and 2.5–20%, respectively [8,14].

Kallala et al. reported, in a study from 2018, symptomatic hypercalcemia in 1 out of 15 patients treated with Stimulan [8]. The incident occurred in a case where 40 cc had been used, which significantly exceeds the upper limit of 20 cc recommended by the manufacturers.

In our study, we encountered complications in one case after using the biocomposite. Granules were later expelled through a fistula, but the area healed without further issues. Palmer et al. described a study showing an overall success rate of 75–100% for two-stage replacement arthroplasty [15]. Although there are many studies reporting success rates of over 90% in two-stage revision, the actual success rate with no recurrence of infection, mechanical failure, or reoperation is 65%.

In our study, we found that the biocomposite, Stimulan, was effective in treating both acute and chronic infections. We decided to keep the implant in 69.73% of the cases, and when Stimulan was applied in 26 cases, 100% of them were cured. Comparatively, the cure rate for cases where Stimulan was not applied was only 57.69%. This demonstrates the effectiveness of Stimulan in treating infections and its benefits in one-stage exchange interventions, where we achieved a 100% cure rate.

Most of the available studies evaluating one-stage exchange arthroplasties have focused on late chronic infections. According to Tsukayama, these infections are responsible for the failure to control the infectious process. Since irrigation and debridement have been linked to high reinfection rates in patients with late chronic infections, exchange arthroplasty is theoretically the most suitable treatment. However, there is a lack of comparative studies in the literature that review DAIR (debridement, antibiotics, and implant retention), one-stage exchange, and two-stage exchange in both acute and chronic infections [16].

In a study by Hansen et al., the authors included 27 patients who underwent one-stage cementless exchange for acute infections for 6 weeks after the initial procedure [17]. Aiming to retain the originally fitted prostheses, the infection control rate was 70% at a mean follow-up of 51 months, but this figure included two patients with Methicillin-resistant *Staphylococcus aureus* (MRSA). The study concluded that despite the potential benefits of using available primary implants, the outcome is still inferior to a one-stage procedure using cemented prostheses, which provides a success rate of approximately 80%.

Van Dijk et al. evaluated the long-term results of revision procedures related to periprosthetic infections with a mean follow-up of 53 months [18]. The overall infection control rate was 84% (107 of 128 cases), of which 18 of 21 cases were successfully treated with a one-stage revision procedure and 89 of 107 cases (83%) treated with a two-stage revision.

When functional outcome is also considered, a systemic review by Leonard et al. showed that one-stage revision surgery was superior. Infection control also includes infection-free survival of the implant [19]. Median infection-free survival of prostheses placed with one-stage revision was 90 months, and for a two-stage revision procedure, 98 months, with an equal follow-up time. Performing a two-stage revision procedure is the most commonly used method to treat chronic periprosthetic joint infections. However, the use of one-stage revision is gaining more and more support.

The current study tried to achieve a per primam cure in the DAIR intervention by applying Stimulan to the site of the infection, which we also achieved with the two-stage exchange interventions where we used the biocomposite and obtained a 42.22% cure, but according to Barton et al., two-stage exchange interventions for arthroplasty infections for both the hip and the knee have a high death rate (24%) [20].

Another study conducted by Steinicke et al. concluded that the success rate after a two-stage revision is low. The study recommends that patients be treated on a case-by-case basis, taking into consideration the specifics of each case [21]. The referenced study involved eight patients with chronic cases, one of whom had oropharyngeal neoplasm and multiple associated comorbidities and unfortunately passed away. Another study conducted by Dimitrios S. performs a retrospective analysis of cases of infection after hip arthroplasty comparing one-stage vs. two-stage procedures over a period of 12 years [18]. They compared the infection eradication rate, the complications, the operating time, the hospitalization period, the intraoperative bleeding, and the functional scores between these two procedures and found that the future “new gold standard” may be surgical intervention in a single stage.

Another important consideration is the duration of hospitalization and the costs associated with the two-stage treatment. In a study published by Blom et al. in 2022, the results of patient-reported outcomes for revision surgeries performed in both one and two stages, as well as the costs of these procedures, were presented [22]. The study concluded that patients prefer early restoration of functionality, the benefits of undergoing a revision surgery in a single stage, and the lower costs associated with this approach compared to the two-stage procedure. These findings contradict the study that demonstrated lower overall costs for the one-stage treatment, as well as better restoration of patient functionality following this intervention.

Studies that have directly compared the efficacy of DAIR versus two-stage exchange for treating PJI have produced mixed results. Zhang et al. found a 70% success rate for DAIR and 75% for the two-stage group, with no statistically significant difference between them [23]. Barry et al. reported that DAIR was equally effective as two-stage exchange in preventing reoperation for infection and more effective in maintaining function [24]. Leta’s team compared DAIR, one-stage, and two-stage revisions and observed re-revision rates for infection of 19% (63/329), 13.9% (10/72), and 11.5% (28/243), respectively [25].

Our study demonstrates the effectiveness of the treatment through DAIR interventions with the application of Stimulan at the site of the infection. Both for cases of acute infection and for cases of chronic infection, where it was decided to keep the primary implant and to apply Stimulan to the site of infection after thorough debridement and irrigation, the healing rate was high. This was not favored by the length of time until the onset of the infection, but by the particularity of the case.

After undergoing hip arthroplasty, patients who require surgery for infection face a 24% risk of death. The two-stage operative protocol has a success rate of 32%, but there is still a 15% risk of reinfection within 4.5 years for those who complete the protocol. The two-stage revision intervention is commonly used to treat chronic infections, but it can be disabling for the patient and has a low rate of functionality recovery as well as an increased risk of death. Because of this, the current trend is shifting towards single-stage surgical interventions [26].

Among the limitations of this study, we can mention the relatively small number of patients, which could have influenced the results.

The very high acquisition costs of this product have made it not always available in the hospital, which is why it was only used during the periods when it could be purchased. This aspect made some of the characteristics of the Stimulan versus non-Stimulan groups different.

## 5. Conclusions

An infectious process correctly detected, diagnosed, and managed by adequate debridement and irrigation and the application of Stimulan granules at the site of infection shows a good healing rate.

The patients with Stimulan had a longer average length of hospitalization, almost half of them required intervention in two periods, and a quarter required the implantation of spacers. However, the cure rate was higher, only in three people could we not control the infection, and there was no death.

Our study confirms that the application of a local antibiotic biocomposite, in addition to the classic treatment of prosthetic hip infections, increases the healing rate for both acute and chronic cases, without adverse effects and complications.

## Figures and Tables

**Table 1:** Characteristics of the study group.

	Total Group (*n* = 76)	With Stimulan (*n* = 45)	Without Stimulan (*n* = 31)	*p*
**Age**, years (mean ± SD)	67.84 ± 12.04	66.66 ± 11.90	69.09 ± 12.20	0.38
**Group age**, *n* (%)				
20–39	2 (2.64%)	1 (2.22%)	1 (3.22%)	0.29
40–59	13 (17.10%)	10 (22.22%)	3 (9.68%)	
60–79	51 (67.10%)	29 (64.44%)	22 (70.96%)	
≥80	10 (13.16%)	5 (11.11%)	5 (16.14%)	
**Gender**, *n* (%)				
Men	50 (65.79%)	30 (66.67%)	20 (64.51%)	0.84
Women	26 (34.21%)	15 (33.33%)	11 (35.49%)	
**Living environment**, *n* (%)				
Rural	33 (43.4%)	18 (40%)	15 (48.38%)	0.39
Urban	43 (56.6%)	27 (60%)	16 (51.62%)	
**Body Mass Index**, *n* (%)				
Normal weight	10 (13.16%)	9 (20%)	1 (3.22%)	0.06
Overweight	50 (65.79%)	28 (62.22%)	22 (70.96%)	
Obesity I	16 (21.05%)	8 (17.78%)	8 (25.82%)	
**Type of infection**, *n* (%)				
Early	21 (27.63%)	12 (26.67%)	9 (29.03%)	0.61
Delayed	35 (46.05%)	20 (44.44%)	15 (48.39%)	
Late	20 (26.32%)	13 (28.89%)	7 (22.58%)	

**Table 2:** Surgical characteristics of patients with Stimulan versus without Stimulan.

	Total Group (*n* = 76)	With Stimulan (*n* = 45)	Without Stimulan (*n* = 31)	*p*
Duration between primary surgery and infection, days (median (25th, 75th percentile))	360 (112.5; 810)	420 (180; 720)	360 (105; 900)	0.80
Previous antibiotic treatment, *n* (%)	13 (17.10%)	8 (17.78%)	5 (16.14%)	0.42
Type of intervention, *n* (%)				
DAIR	52 (69.73%)	26 (57.78%)	26 (83.83%)	0.01
Two-stage	24 (20.27%)	19 (42.22%)	5 (16.14%)	
Reimplantation of the prosthesis, *n* (%)	16 (21.05%)	10 (25.64%)	6 (16.21%)	0.32
Spacer implantation, *n* (%)	13 (17.10%)	12 (22.66%)	1 (3.22%)	0.007
Hospitalization, days, (median (25th, 75th percentile))	15 (10; 33)	22 (11; 51)	13 (10; 22)	0.16
Evolution, *n* (%)				
Cured	52 (68.42%)	42 (93.33%)	10 (32.25%)	<0.00001
Active	16 (21.05%)	3 (6.66%)	13 (41.94%)	
Deceased	8 (10.53%)	0 (0%)	8 (25.81%)	

## Data Availability

The data published in this research are available on request from the first author and corresponding authors.
